# Emotional pain in non-human mammals: from neural circuitry and opioid signaling to dysthymia-like states, grief-like behaviors, and physiological dysregulation

**DOI:** 10.3389/fvets.2026.1878991

**Published:** 2026-07-29

**Authors:** Daniel Mota-Rojas, Alexandra L. Whittaker, Genaro A. Coria-Avila, Ismael Hernández-Avalos, Julio Martínez-Burnes, Adriana Domínguez-Oliva, Adriana Olmos-Hernández, Fabiola Torres-Bernal, Brenda Reyes-Sotelo, Agatha Miranda-Cortes, Alejandro Casas-Alvarado, Temple Grandin

**Affiliations:** 1 Neurophysiology, Behavior, and Animal Welfare Assessment, DPAA, Universidad Autónoma Metropolitana (UAM), Mexico City, Mexico; 2 School of Animal and Veterinary Sciences, University of Adelaide, Roseworthy Campus, Roseworthy, SA, Australia; 3 Instituto de Investigaciones Cerebrales, Universidad Veracruzana, Xalapa, Mexico; 4 Department of Biological Sciences, Clinical Pharmacology and Veterinary Anesthesia, FESC, Universidad Nacional Autónoma de México, Cuautitlán, Mexico; 5 Facultad de Medicina Veterinaria y Zootecnia, Instituto de Ecología Aplicada, Universidad Autónoma de Tamaulipas, Victoria City, Mexico; 6 Department Bioterio and Experimental Surgery, Instituto Nacional de Rehabilitación Luis Guillermo Ibarra Ibarra (INR-LGII), Mexico City, Mexico; 7 Department of Animal Science, Colorado State University, Fort Collins, CO, United States

**Keywords:** dysthymia, emotional pain, grief-like behavior, panic, thanatological behaviors

## Abstract

This review aims to analyze the neurobiological, pathophysiological, and ethological foundations of emotional pain in non-human mammals, with particular emphasis on the transition from adaptive responses to pathological states such as animal dysthymia and Takotsubo syndrome. In the review, four conditions that can activate emotional systems will be discussed, such as pain derived from surgery or injury, stressful conditions that do not cause pain (e.g., unknown noises or smells), separation stress where an animal is isolated from the group (e.g., dam-calf separation), and death of a familiar animal or conspecific. The review will examine the role of subcortical brain regions, highlighting the amygdala—particularly the basolateral complex and central nucleus—as key integrators of negative affect and stress responses. In addition, it will address the contribution of cortical regions, such as the anterior cingulate cortex, and their interaction with the endogenous opioid system in the processing of affective suffering. The review will also incorporate evidence of thanatological behaviors and mourning-like responses, suggesting a degree of death awareness and social sensitivity to loss across multiple species. While the function of these neural circuits is fundamentally adaptive, chronic activation may induce neuroplastic changes and neurotransmitter dysregulation, leading to maladaptive outcomes. These include persistent anxiety, depression-like states, thanatological behaviors, and stress-related cardiac conditions such as Takotsubo syndrome, all of which may compromise animal welfare. Finally, further comparative research is needed to deepen our understanding of emotional pain in non-human animals.

## 1 Introduction

Experiencing emotional pain is fundamentally adaptive and serves a crucial evolutionary function: it signals social loss, separation from attachment figures, or threats to social bonds, thereby motivating behaviors aimed at restoring proximity and maintaining social connections. In this sense, emotional pain acts as an “alarm system” that promotes survival and social cohesion. However, when emotional pain becomes chronic, overwhelming, or disproportionate to the triggering event, it can transition from an adaptive response to a maladaptive state, contributing to psychopathological conditions. The transition from an adaptive to a pathological response becomes evident in specific conditions, such as unresolved grief, separation anxiety disorders, or dysthymia-like states.

In humans, psychological or emotional pain is self-reported and defined as “an unpleasant, overwhelming, and upsetting internal experience,” often manifested as distress, anxiety, and depression (1). This aversive affective state is commonly associated with social separation, loss, and disruption of attachment bonds (2–5). It also relates to grief and mourning—defined as an intense but transient emotional reaction to the permanent loss of a close bond (6), as well as dysthymia—a persistent and chronic state of depression (7). In non-human animals, assessing emotional pain remains challenging due to the absence of verbal self-reports. Nevertheless, animals exhibit behavioral and physiological responses indicative of aversive conditions. According to Panksepp, these responses can be interpreted through a framework of “critical anthropomorphism,” which allows for cautious inferences about affective experiences based on shared neurobiological substrates across species (2, 8). Thus, emotional suffering in animals—as inferred from experimental studies—encompasses states associated with fear, loneliness, boredom, frustration, helplessness, grief-like responses, depression, and pain (9). Indeed, emotional and physical pain partially share neural pathways across species, involving both subcortical and cortical regions (1, 10–12).

Empirical evidence has documented overlapping neural substrates in basic affective circuits across all mammalian brains and, to some extent, in birds. Within Panksepp’s seven basic emotional systems, those with negative valence, such as PANIC/GRIEF, FEAR, and RAGE, require contributions of different subcortical brain areas located subcortically, but modulated cortically (2). These negative affective states involve partially shared neural circuits (3). Panksepp has mentioned that, in this context, pain involves two general domains: (i) sensory-discriminative component and (ii) affective-motivational component (4, 13). In humans, the PANIC/GRIEF system is associated with psychological pain or pain related to social loss when individuals feel attachment loss or separation distress (5, 14). According to the social pain framework, physical and social pain rely on partially overlapping neural substrates (e.g., the brainstem, diencephalon, and certain cortical regions) rather than identical neurobiological pathways (3).

For instance, in infant macaques subjected to social pain distress, damage to the dorsal or ventral region of the anterior cingulate cortex (ACC) significantly decreases the expression of distress vocalizations (<10 per minute vs. >20 p/min in intact macaques), a behavior that can be emitted by infant mammals (and birds) during isolation from their mothers (13, 15). Certainly, human and animal neuroimaging studies of social pain have reported similar findings: social rejection during social dynamics such as play leads to higher activation of the ACC and insular cortex in response to social distress (13, 16, 17). However, this type of evidence does not necessarily imply that emotional pain originates within cortical regions; it may suggest that cortical regions—such as the ACC—modulate the expression of affective states that originate subcortically. For instance, the ACC is also activated during physical pain, and the physical-social pain overlap could be considered within the pathophysiological framework of emotional dysfunctions, such as dysthymia-like states (3, 12), separation anxiety, and grief-like states in non-human animals (16).

During dysthymia-like responses in animals, which manifest as chronic over-activation of affective circuits (18), the ACC and insula are brain regions involved in integrating and processing social stimuli and emotional pain within the chronic depressed mood circuit (16, 19). Unlike acute stress, the term dysthymia is used in a translational sense to describe persistent depression-like affective states observed in animal models—rather than as a direct diagnostic equivalent of the human disorder—and is characterized by anhedonia and extreme sensitivity to rejection (12). In humans, dysthymic disorder arises from altered connectivity and cortical top-down modulation (7), which alters reward and punishment monitoring (20, 21) and promotes persistent affective pain in affected individuals. Functional changes in the ACC, amygdala, insula, and certain diencephalic nuclei may maintain the brain in a depressed state regardless of external stimuli, thereby depleting motivational resources and consolidating dysfunction in reward processing (12, 22). The severity of these functional brain changes could be related to the brain-heart axis, in which a persistent emotional stress has been associated with autonomic dysregulation and increased catecholamine release, which may contribute to stress-induced cardiomyopathy, such as Takotsubo syndrome (23, 24). This suggests that persistent depressive disorders and chronic pain not only reconfigure the neuro-circuits of the affective component of pain processing (12) but also cause destabilization of autonomic homeostasis.

Understanding these neuroanatomical substrates is essential for deciphering how experiences of social separation become consolidated as persistent emotional pain. In this sense, social and affective suffering constitutes an emotional experience in social mammals, emerging from the threat, rupture, or loss of bonds, whether with conspecifics or attachment figures. Although these experiences are well-documented in humans and are associated with behavioral, neurobiological, and physiological alterations (15, 25, 26), studies in non-human animals are limited. Comparative affective neuroscience suggests that several mammalian species share evolutionarily conserved emotional systems involved in attachment, separation distress, and social bonding, supporting the possibility that animals experience negative affective states related to loss and social disruption (5, 27).

**Figure 1:**
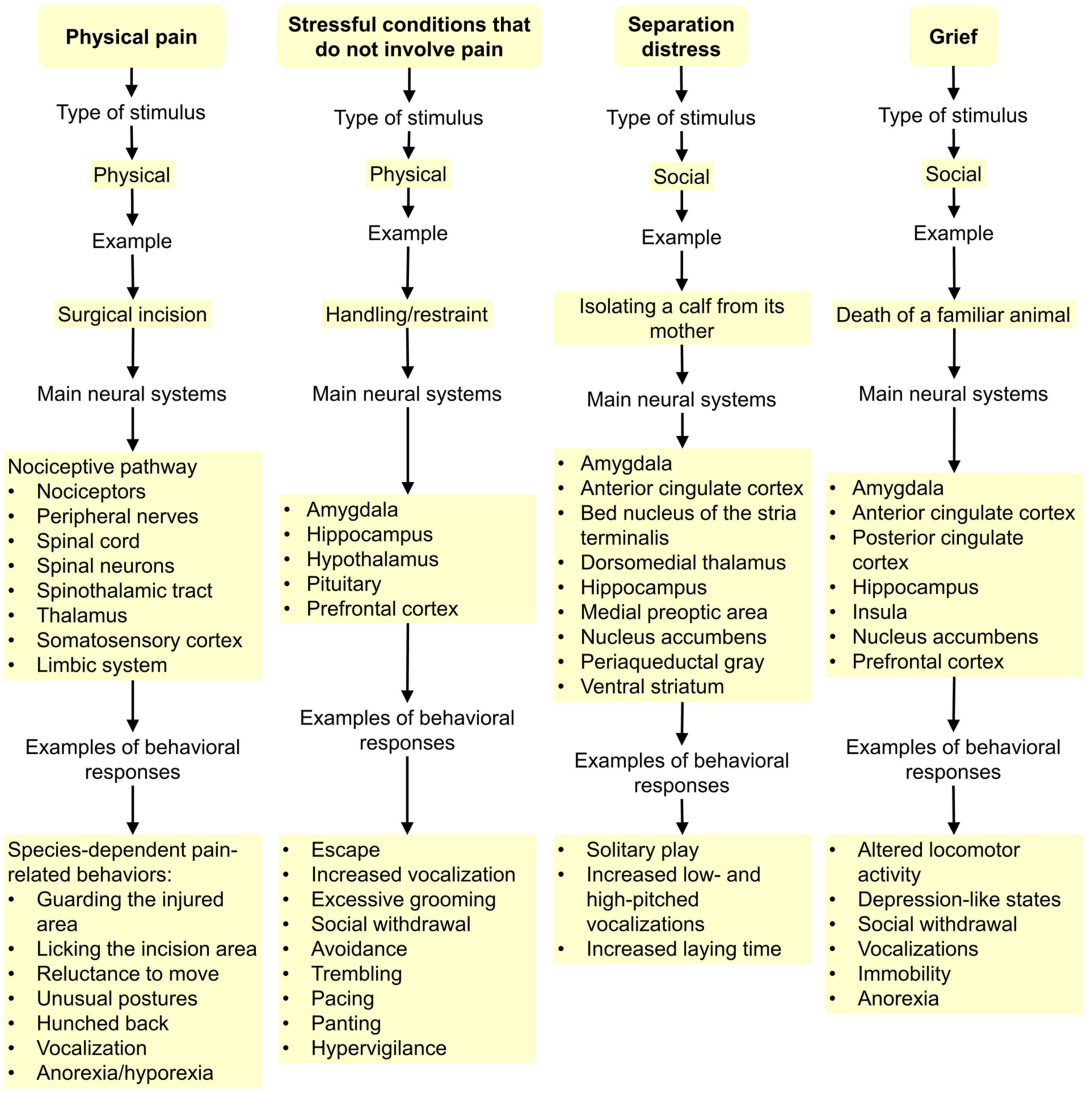
Examples of conditions that can activate emotional systems.

Within this context, grief, separation anxiety, and physiological responses derived from emotional stress represent expressions that are related to affective suffering in humans (6, 28). That is why their recognition allows for the reduction of persistent negative affective states (29–31) that can become sources of prolonged suffering and emotional dysregulation. Despite increasing evidence on social pain and affective processing in animals, the neurobiological mechanisms underlying persistent emotional distress and its pathological consequences remain poorly integrated across disciplines. This review aims to examine these mechanisms from a neurobiological, ethological, and pathophysiological perspective, addressing conditions that can activate emotional systems (e.g., physical pain, stress, social separation, and death of a conspecific) (Figure 1) (32–37). The manuscript will also address the role of the anterior cingulate cortex and the endogenous opioid system in the processing of affective suffering. It will also include scientific evidence of thanatological behaviors and mourning rituals that suggest a notion of death and social sensitivity to loss across various species.

## 2 Neurobiology of animal dysthymia-like states

Dysthymia is derived from the Greek words “*dys-*”, meaning “ill” or “bad”, and “*-thymia*”, meaning “emotions”, representing a persistent depressive disorder (7). In humans, it is characterized by social-motivational alterations, including anhedonia, depressed mood, extreme fatigue, and persistent rejection sensitivity (38). According to Panksepp and Biven (14), the PANIC/GRIEF system in mammals arises primarily from ancient subcortical circuits rather than the neocortex. The key evidence comes from electrical stimulation experiments in animals in the 1980’s, where electrodes implanted in deep subcortical regions such as the periaqueductal gray (PAG), the dorsomedial thalamus (DMT), the ventral septal area, and the bed nucleus of the stria terminalis (BNST), evoked full-blown vocalizations even in decorticated individuals (those without a neocortex), a behavior frequently used as an indicator of distress in non-human primates related to injury or death of a group member (39, 40). However, although animal vocalizations have been associated with the death of a conspecific (41) and may be an important indicator of separation distress or activation of the PANIC/GRIEF system in mammals, it should not be treated as interchangeable evidence for grief, depression-like, dysthymia-like, or anhedonia-like states without further qualification.

**Figure 2:**
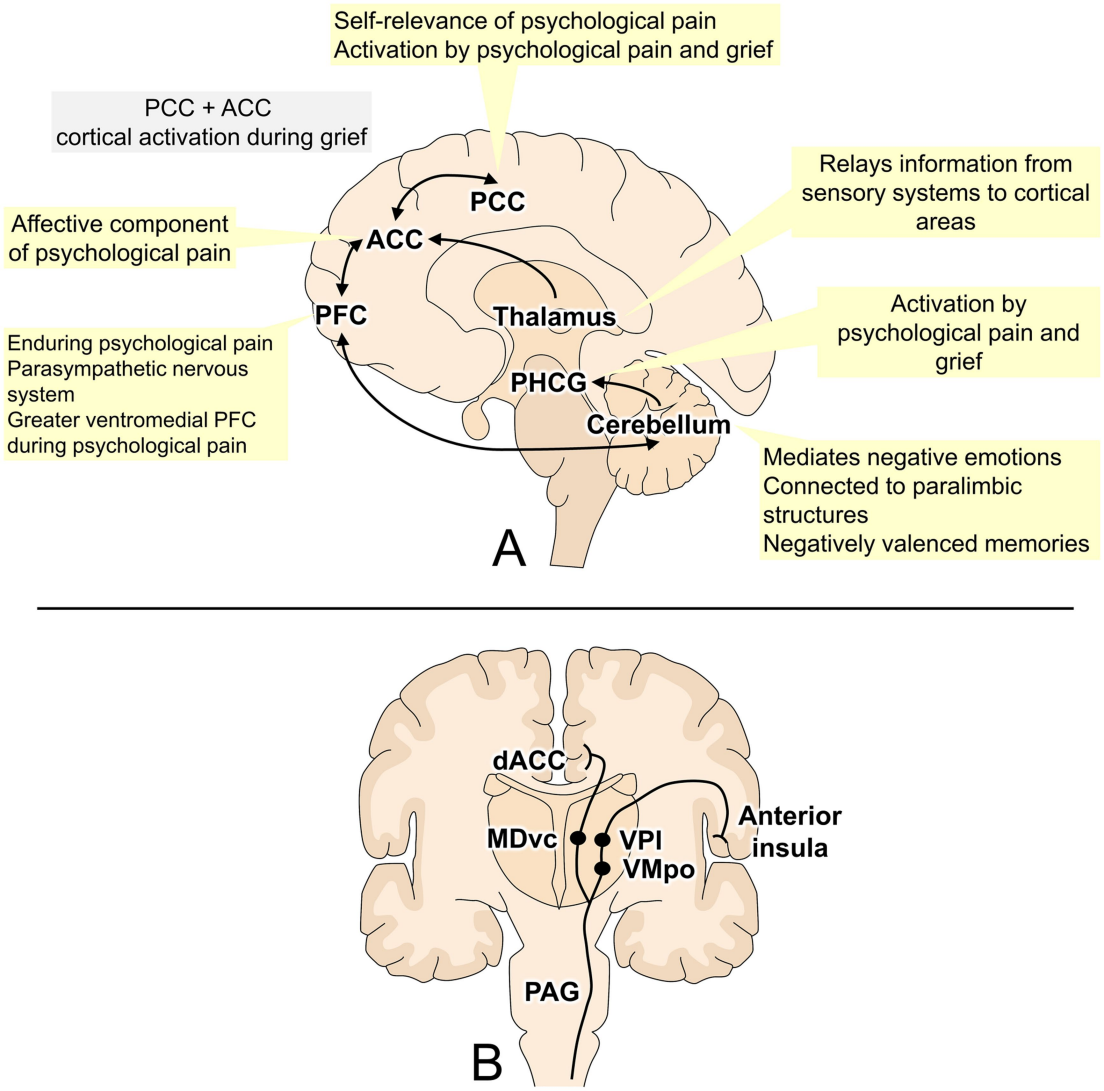
Human brain structures that participate in emotional pain and suffering. **(A)** Neural networks for processing psychological or emotional pain. A tentative proposed network includes structures such as the ACC, which provide the affective component of psychological pain and the PCC, an area that shows activation during grief, fear, and sadness (5, 165). The cerebellum, PFC, PHCG, and thalamus also participate in mediating negative emotions and relaying information to other brain structures. Double-headed arrows indicate bidirectional communication with both brain structures. **(B)** Brain areas activated during pain-related suffering. As shown in the diagram, brain regions involved in psychological pain partially overlap with nociceptive pain networks. Whether these projections are similar in non-human animals remains l under study. ACC, anterior cingulate cortex; dACC, dorsal anterior cingulate cortex; MDvc, ventral caudal portion of the medial dorsal nucleus of the thalamus; PCC, posterior cingulate cortex; PFC, prefrontal cortex; PHCG, parahippocampal gyrus; VMpo, posterior part of the ventral medial nucleus of the thalamus; VPI, ventral posterolateral nucleus of the thalamus.

These findings suggest that emotional processing mainly relies on subcortical activity. However, cortical regions (e.g., the ACC), appear to also participate in modulating emotional processing. This has been reported in humans when deep brain stimulation is applied to the ACC, inducing a state of “depressive despair” that disappears when stimulation ceases (42). Similarly, PET imaging of humans experiencing profound sadness shows robust activation of the ACC alongside subcortical structures. Thus, evidence in humans suggests that the ACC serves as a higher-order regulatory and representational node, whereas subcortical circuits generate the core, raw affective experience of grief. Figure 2 schematizes the main human brain structures that participate in negative emotions, such as suffering and emotional pain (3, 16). In non-human animals, a definitive neural pathway has not been established, as studying emotions is challenging. Nonetheless, animal experimental evidence suggests that brain region activation may align with that in humans during emotional processing, as discussed below.

In humans and non-human animals, the corticolimbic system has been reported to integrate affective processing, social behavior, and autonomic regulation system (12, 16, 43). The corticolimbic system is formed primarily by the prefrontal cortex (PFC), amygdala, hippocampus, anterior insula cortex (IC), and ACC (44). The role of the corticolimbic system in the processing of negative emotions has been studied in male Wistar rats exposed to aversive audiogenic stress (45). Using computed tomography imaging, it was found that the activity of the ventral hippocampus, amygdala, and PAG increased significantly when listening to the acoustic aversive stimulus. In the same species, increased c-Fos expression—a marker for neuronal activation—in the hippocampal ventral subiculum, ventral lateral septum, and lateral hypothalamus tuberal nucleus was found when exposing male Sprague–Dawley rats to emotional stress (elicited by restraint stress, bright light, and other stressors) (46). Disgust-related states in Macaque monkeys have been described as labeled connections among the IC, the orbitofrontal cortex (areas 11, 12, and 13), and subcortical structures (e.g., the putamen, the posterior part of lateral hypothalamic area, and the ventral tegmental area [VTA]) (43). The effects of depressive-like states on corticolimbic activity have also been described in animal models (47). In male and female Long-Evans rats subjected to maternal maltreatment, depression-like behaviors (e.g., deficits in social behavior) were associated with attenuation in amygdala activation (lateral and basal nuclei) (48). In the same study, depressive-like states in rats dampened c-Fos expression in the PFC (including the ACC, prelimbic and infralimbic cortices), and the nucleus accumbens (NAc) was also reported (48). In mice, Yuan et al. (49) reported that depressive-like states are mediated by inhibition of the pathway connecting the AAC to the basolateral amygdala (BLA). Furthermore, a systematic review of 43 rodent models of depression concluded that, through opto-and chemogenetic methodologies, the central circuitry for depressive-like behavior in the species is characterized by decreased function of the medial PFC and NAc, hyperactivity of VTA, and participation of pathways involving the ACC, amygdala, and hippocampus (50). However, the authors emphasize that the neurocircuitry underlying depressive-like states in animals is complex, and the structures studied are only a subset of the neural network.

In humans, it is suggested that the failure of inhibitory control—or hyperactivity—of ACC facilitates the development and chronicity of major depressive disorders, such as dysthymia (19, 51, 52). In animal models, increased activity within ACC-amygdala circuits has been associated with depression-like behaviors. For example, Huang et al. (53) performed c-Fos mapping in mice with a depressive-like state associated with orthotopic colorectal cancer. The findings indicated a significant and positive correlation between c-Fos expression in the ACC and depressive-like behaviors, such as reduced grooming and social interaction (*r* = from 0.52 to 0.87). Moreover, the authors reported that inhibition of the ACC activity reduced depression-like behaviors (53). Similarly, in a mouse model of depression subjected to maternal separation and restraint stress, it was reported that aberrant signaling in the PFC-amygdala-hypothalamus circuit was associated with depressive- and anxiety-like behaviors (54). Becker et al. (55) also reported hyperactivity of the BLA-ACC pathway in mice exhibiting depressive-like behaviors associated with induced neuropathic pain. Hyperactivity was observed as increased c-Fos immunoreactivity (378.2 ± 49.10 cells in injured animals vs. 309.3 ± 47.60 cells in sham mice) in BLA neurons projecting to the ACC. Increased immunoreactivity was accompanied by depressive-like behaviors, including a significant reduction in grooming time (injured: 80.67 ± 8.22 s vs. sham: 125.90 ± 10.80 s). The authors also reported that inhibition of the BLA-ACC neurocircuit, using a green light-emitting laser, reversed depression-like behaviors after 7 weeks. Conversely, in non-human primates, electrical stimulation of ACC caused the spontaneous manifestation of distress vocalizations (16).

The regulatory role of the ACC in emotion and social behavior has been observed in cynomolgus macaques with bilateral cingulate cortex lesions. Lesioned animals exhibited a significant reduction in the number of social interactions (physical contact) (20 per 10 min), activity level (40% proportion of time), and number of vocalizations (<5 per 10 min) compared to control individuals (60 per 10 min, >60%, and >10 per 10 min, respectively) (13), indicating a role in social behavior and emotion. Likewise, squirrel monkeys subjected to cortical ablations showed a significant decrease in the number of isolation calls (from >10 to 0 per minute), a behavior associated with social distress (17). These findings suggest an important role for this region in affective and social processing.

### 2.1 The endogenous opioid system: neurochemistry of separation

**Figure 3:**
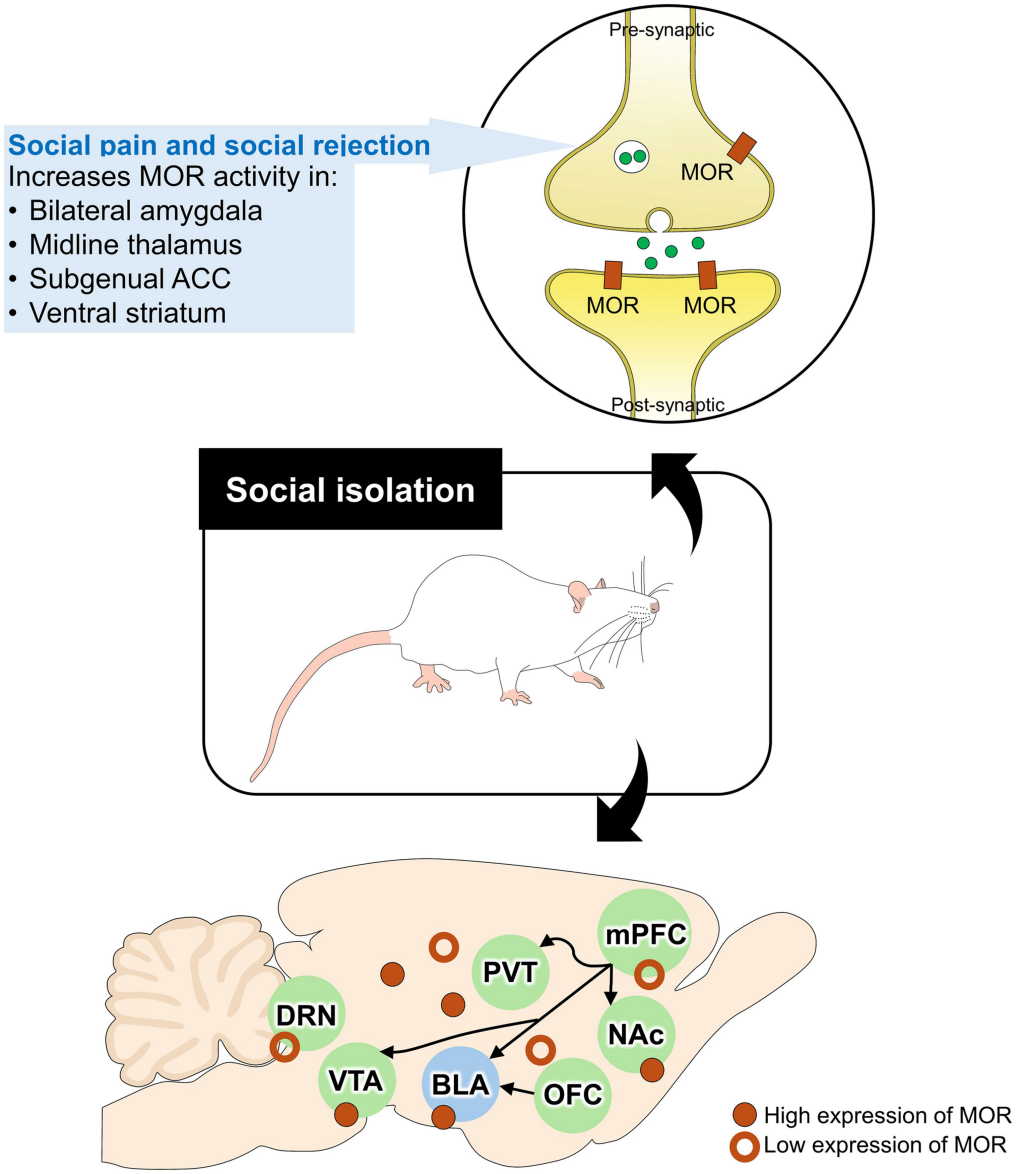
Social isolation and *μ*-opioid receptor activity. In murine models, social isolation has been observed to be mediated by hypo-activation of brain areas marked by green circles (e.g., mPFC, NAc, and VTA) (166), and hyper-activation of the BLA (marked with a blue circle) (167). In most of these regions, high MOR expression modulates social pain and attachment. During social rejection, MOR activity increases in the amygdala, midline thalamus, subgenual ACC, and ventral striatum. ACC, anterior cingulate cortex; BLA, basolateral amygdala; DRN, dorsal raphe nucleus; mPFC, medial prefrontal cortex; MOR, μ-opioid receptor; NAc, nucleus accumbens; OFC, orbitofrontal cortex; PVT, paraventricular thalamus; VTA, ventral tegmental area.

The endogenous opioid system, particularly *μ*-opioid receptors (OPRM1) in the dorsal striatum axis, has been shown to modulate separation distress and social attachment in human and animal models (56), suggesting a role in regulating negative affective states related to social loss or pain (Figure 3). Under homeostasis, social interaction and play stimulate the release of *β*-endorphins which act as natural modulators of distress related to social separation or of pain relief (57, 58). In infant guinea pigs subjected to social isolation, the administration of low doses of morphine significantly decreased the frequency of distress vocalizations to about 6% of baseline values, In comparison, the administration of naloxone (an opioid antagonist) had the opposite effect, significantly increasing distress vocalizations by 52% (59). This has also been observed in primates (60) and rodents (61).

Thus, opioids appear to be key neuromodulators for physical and psychological pain (3). In animals (mice and non-human primates), it has been suggested that low availability of these peptides or absence or variation of the OPRM1 receptor might be associated with reduced social bonding, nociceptive pain, and distress (3, 16, 62, 63). In humans carrying the A118G polymorphism in the OPRM1 gene, a gene associated with reduced opioid receptor availability, impaired emotional pain modulation with a tendency toward sensitization was observed (64). Thus, alterations in the endogenous opioid system may represent an important substrate involved in affective processing of pain (Figure 3).

### 2.2 Chronic pain leads to neurocircuitry alterations and emotional disorders

Chronic stress and persistent pain animal models have been associated with mood disorders, including depressive-like states (65). These animal models have shown functional and synaptic changes in the ACC and related cortical and subcortical brain regions (66, 67), which may provide a neurobiological framework for prolonged negative affective states. For example, in a rat model of chronic pain (intra-articular injections of Freund’s Complete Adjuvant), magnetic resonance imaging showed that lesioned animals exhibited a significantly increased functional connectivity of the ACC with the somatosensory cortex, basal forebrain region, hypothalamic region, and the bed nucleus of the stria terminalis (66). In male mice subjected to nerve injury, Sellmeijer et al. (65) found an increased firing rate in the AAC (areas 24a and 24b), reflecting electrophysiological hyperactivity of the AAC. Similarly, in a murine model of chronic stress-induced hyperalgesia, it was reported that stressed rats exhibited increased activity in the rostral ACC and pathway synchrony between the ACC and the BLA (68). These studies address roles of cortical and subcortical areas in the processing of negative emotions.

In animal models and humans, persistent pain also induces abnormal long-term potentiation (LTP) in adult glutamatergic synapses in the ACC and IC (12). LTP is the basic mechanism of learning and memory, involving the strengthening of a connection between two neurons after repeated glutamatergic activation (69). Therefore, during chronic pain, the ACC and IC may contribute to the potentiation of emotional distress learning through synapses optimized during the transmission of distress signals, which activate with minimal stimuli or even without them.

At the molecular level, this strengthening of chronic pain neurocircuits has been associated with three main modifications (70). First, an increase in the density and sensitivity of postsynaptic N-methyl-D-aspartate (NMDA) receptors in amygdala neurons facilitates an excitatory response to minimal environmental stimuli. These findings have been observed in adult rats subjected to NMDA blockade, which report disruption of conditioned aversion and inhibition of long-term potentiation (LTP) in IC (71), and suggest that NMDA receptor upregulation sensitizes emotion-management centers and promotes pain-induced emotional disorders, similar to the well-established mechanisms of memory formation. Thus, NMDA receptors may play an important role within the neurocircuits involved in the affective component of pain and depressive-like states.

Furthermore, it is proposed that dysfunction of cortical GABAergic inhibitory interneurons in the ACC could contribute to disinhibition of the amygdala (12). Studies in adult male rats have shown that chronic stress reduces the density of calbindin-positive GABAergic neurons by 40–50% in the orbitofrontal cortex, a key brain region that appears to be implicated in the affective dimension of pain (72). Conversely, stress-resilient rats showed a significant increase in the density of cholecystokinin neurons (+31%) and neuropeptide Y neurons (+7%) (73). This is similar to what was observed in prefrontal GABAergic networks, including those l in the ACC (74). This evidence suggests that the integrity of GABAergic networks and degeneration in the ACC could contribute to overriding affective compensation mechanisms, thereby, consolidating the emotional dysfunction state.

Additionally, long-term depression (LTD), one of the normal mechanisms that should restrict memories of fear or chronic stress, exhibits impairment, preventing the animal from compensating for the negative affective response (12). A mouse model of neuropathic pain showed that, following chronic constriction injury of the sciatic nerve, the ACC’s ability to induce LTD rapidly decreases and eventually disappears, and even after recovery, the impairment persists (75). Therefore, these findings could explain the persistence of two components of pain even when the initial cause has disappeared.

This synaptic strengthening of the ACC-Amygdala pathways may stabilize affective hypervigilance and social avoidance response (12). These findings suggest that long-term plasticity changes in ACC–amygdala circuits may contribute to the persistence of negative or depression-like affective states even after the original stressor has disappeared. Nevertheless, the neurocircuits, neuromodulators, and neurophysiological alterations involved in the affective component of pain and depression disorders remain unclear in both humans and animal models.

## 3 Social and affective suffering

In social mammals, both human and non-human, interactions regulate physiological and behavioral activity. Social disruption has been associated with stress-related behavioral and physiological changes (76).

### 3.1 Grief-like states and mourning: scientific evidence

Grief-like states, mourning, and bereavement behaviors in non-human animals are understudied topics (28, 77). Some researchers are still hesitant to attribute complex emotions to non-human animals (78). It is important to establish the differences between grief, separation distress, and mourning to understand and connect these complex emotions. In humans, grief is a complex and intense emotion characterized as a reaction to the permanent rupture of a strong social or familial bond. It is processed by the emotional system after recognition of irreversible loss, a process triggered by neurochemicals associated with social attachment and bonding (27). Averill (30) and Pollock (77) mention that grief is a biological component of bereavement with phylogenetic evolutionary origins. In contrast, separation distress, frequently observed in non-human animals, is defined as an acute, involuntary, and temporary isolation event (e.g., dam-calf separation) that might serve as an adaptive mechanism of proximity seeking (79), but not as the irreversible loss of an attachment. Therefore, grief could be an evolution of a stress reaction to separation, helping maintain group cohesion in social species (30). On the other hand, the state of bereavement is described as psychological stress associated with symptoms of depression, anxiety, and anger (15), which persist for weeks or months and sometimes become chronic (80).

Evstigneev et al. (81) note that the neural systems involved in human grief can be divided into six domains: emotion regulation; reward processing and attachment; memory and cognitive control; executive function; structural brain changes; neuro-immune and neuroendocrine interactions, and attentional dysregulation. The first factor induces greater activation across brain structures, accompanied by persistent hyper-connectivity (82, 83). Second, reward processing and attachment mechanisms lead to hyper-activation of the nucleus accumbens when the deceased individual is viewed, with increased connectivity within the ventral striatum during early intense grief (84). Third, memory, cognitive control, and executive function are impaired by grief and pain-related cues (85). Fourth, structural brain changes are present in the hippocampus, amygdala, and supramarginal gyrus (86). Thus, neuro-immune and autonomic interactions reflected in correlations between activity and elevated pro-inflammatory cytokine levels, along with stress-sensitive changes (87). Finally, attentional dysregulation is characterized by involvement of the fronto-parietal network, with the severity of grief being linked to greater attentional interference and slower processing of information related to the loss (37).

In this section, the terms grief-like behaviors and mourning-like responses are used in a comparative sense to describe observable behavioral and physiological responses to social loss in animals. However, while evidence in humans might provide a useful comparative framework, it cannot be interpreted as direct evidence for grief-related cognitive or fronto-parietal changes in non-human animals.

Human affective neuroscience suggests the existence of evolutionarily conserved emotional systems involved in attachment and separation distress. Brain mapping using electrical stimulation of the separation distress system has identified circuits from the dorsal PAG to the ACC (5, 88). These circuits are activated by glutamate and corticotropin-releasing factors. In contrast, they are inhibited by endogenous opioids, oxytocin, and prolactin. It is suggested that these circuits are tonically aroused during grief and sadness (2, 27).

In humans, an acute grief response could require the addition of other neuro-affective changes before individuals succumb to sustained depressive lassitude and despair. It has been proposed that cytokines, including interleukin-1, could promote feelings of illness, in addition to endogenous inflammatory cascades, which could lead to decreased seeking arousals (89). This could potentially facilitate the onset of depression in response to stressors (90).

In humans, bereavement has been associated with autonomic and cardiovascular changes, which may offer a useful model for understanding stress-related responses in non-human animals. Buckley et al. (80) reported in human patients that early bereavement (within 2 weeks of death) was associated with an increase in systolic blood pressure (130.3 mmHg) and heart rate (74.0 bpm) compared to individuals without losses (127.5 mmHg), particularly during daytime hours. In contrast in the 6 months following, a reduction in blood pressure was observed in non-hypertensive patients (129.3 mmHg, *p* = 0.22), suggesting that increased hemodynamic forces could contribute to the increased risk of cardiovascular disease associated with loss due to death.

In recent years, negative and positive emotions in animals have gained relevance. It considers the evaluation of both behavior and the physiology of emotional valence (91, 92). Other related aspects include subjective human experiences, assessed through qualitative assessments of animal behavior (93) and cognitive appraisal (or information processing) of ambiguous stimuli such as loss, risk, and aversion tendencies, which could indicate general emotional states (94, 95). This has highlighted potential behavioral, physiological, and neuroanatomical changes associated with separation distress, the loss of a social bond, or the death of a conspecific (5, 31, 96). Mourning-like behavior in animals has been reported in dolphins (97).

**Figure 4:**
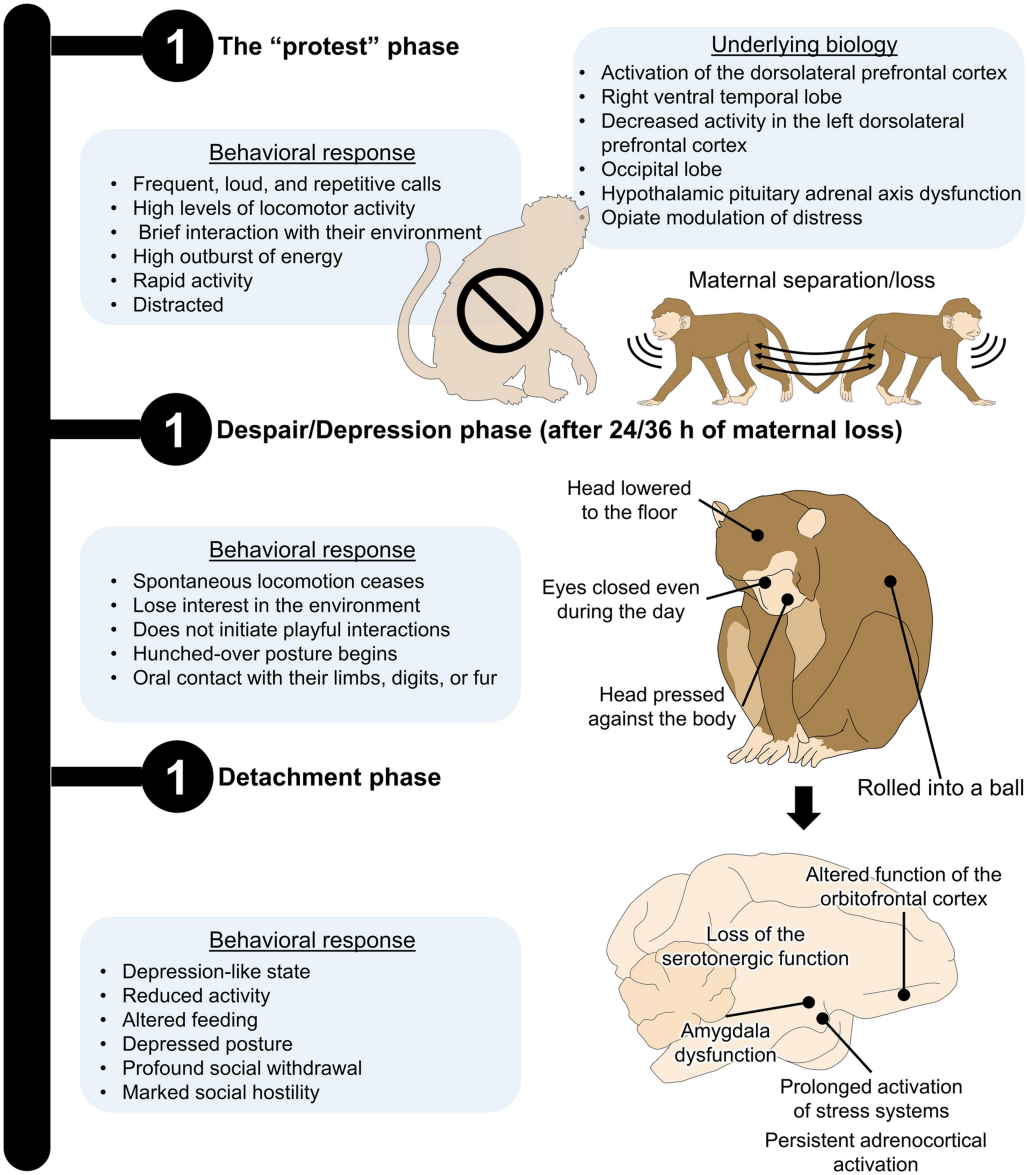
Proposed phases of non-human primates’ reactions to loss (prolonged maternal separation).

In non-human animals, the distress-related responses during prolonged separation and the death of a conspecific are often compared with human grief (78, 98). Studies involving monkeys and apes describe separation-related distress following the bereavement of friends or family members. A two-phase reaction was observed in prolonged, involuntary separation from an attachment figure, in which young monkeys exhibited intense agitation, pacing, and vocalization—behaviors that would lead to reunion with their attachment figure, termed protest behavior (78). Over time, the prolonged separation led the animals to progress from high activity levels to silent immobility, sitting in a hunched posture, and reduced interest in the environment, which was interpreted as the onset of a depressive state or despair (78). This process is schematized in Figure 4 (60, 99–101). These findings suggest that non-human primates can develop a depressive-like state due to prolonged separation rather than a permanent loss. However, a study in Bonnet macaques showed similar behavioral responses of mothers after the loss of their infants (98). Bereaved females received significantly less grooming and initiated hugging significantly less than females with their infants. Moreover, females who lost their infants also displayed significantly more stress-related behaviors, such as yawning, scratching, and woo-calls. These stress-related behaviors are considered among the behaviors observed in the despair stage of grieving (102), which might suggest that animals—at least non-human primates—exhibit grief-like responses to permanent loss.

The studies discussed suggest that following the rupture of a social bond, separation-related responses might be observed in non-human animals. In response to separation distress (prolonged separation but no permanent separation), behaviors of withdrawal, apathy, rejection, hostility, lethargy, and attempts to recover have been observed in farm animals (103, 104) and chimpanzees (105). Some other studies suggest that these experiences of loss have been observed in elephants (106), non-human primates (107), and cats (108).

Caregivers’ perceptions have also been used to identify emotional and behavioral responses related to the death of a conspecific (108–110). In companion dogs, owners using the validated Mourning Dog Questionnaire, reported that following the death of the conspecific, the surviving dog exhibited a reductions in activity and play by 46 and 57%, respectively (111). Increases in sleep (35%), vocalizations (30%), fearfulness (35%), and attention-seeking behaviors (67%) were also reported as grief-related behavioral responses. Veterinarians have also observed changes in animal behavior at the time of conspecific euthanasia, particularly in dogs, although they are rarely observed (22% of respondents) (112). Among these behaviors, 44% said that animals become quiet, 31% reported unusual sounds emitted by the surviving animal, and less than 10% observed agitation. Moreover, it has been observed that caregivers’ grief is associated with increased sleep, solitary behavior, and hiding by companion dogs and cats in response to the loss of another animal (113).

In domestic cats, Greene and Vonk (108) examined predictors of the response of surviving cats after the death of a pet in the same household. The results from 412 cat caregivers, as well as from the 452 surviving cats and their relationships with the deceased pet, were characterized by long-term behavioral changes (increases in sleeping time, time spend alone, and hiding). Of the 452 cats, 281 reacted to the death of another cat, while 171 reacted to the death of a dog. Immediate changes indicated that the caregiver’s attachment strength was the only predictor of attention-seeking behavior. In contrast, the relationship between the companion animals was the only significant predictor of decreased sleep, feeding, and play. Furthermore, activities the animals had performed together before their companions’ deaths elicited greater fear in the surviving cat after its companion’s death. Regarding long-term changes, the strength of the caregiver’s attachment to the surviving animal and the length of time the animals had lived together increased attention-seeking behavior in the weeks and months following death. This is similar to what was observed by Walker et al. (113) in 159 dogs and 152 cats through owner reports, where the most common behavioral changes were affectionate (74% dogs *vs*. 78% cats), with territorial behavior being more prevalent in cats (63%) compared to dogs (60%). Both species demanded more attention and displayed more affiliative behaviors, and spend more time searching for their death companion’s favorite spot (30%). In the case of dogs, they reduced their food consumption by 35% and their eating speed by 31%, but increased their sleep time by 34%. Cats, after the death of their companion, increased the frequency and volume of their vocalizations by 43 and 32%, respectively. In both dogs and cats, the average duration of these changes was less than 6 months.

In wolves, Boyd and Pletscher (114) observed that the mother near her den buried two-week-old dead wolf (*Canis lupus*) pups. In contrast, in the case of *Canis lupus dingo*, dead pups have been observed to be transported to different locations in the days following their death.

Direct evidence regarding grief- and mourning-like states in farm animals is limited, and most research has focused on the behavioral changes to transient separation-distress. This has been reported in Angus-Holstein cross calves (3–6 days old) in whom temporary dam-calf separation (for 60 min) significantly increased lying and sleep-like behavior (~10 bouts during dam-calf separation *vs*. ~7 bouts). This response is frequently associated with negative states and emotional distress (104). In the same species, Mac et al. (115) found that the activity rate of Holstein-Friesian calves, after 3 days of full dam-calf separation, significantly decreased (from 632 min/day to 467 min/day).

In Dickinson and Hoffmann’s (112) study, although most veterinarians explained that the behavioral responses of animals to the loss of a conspecific respond to “animal grief and empathy,” these concepts are challenging to establish in non-human animals. Moreover, behaviors such as reduced activity fearfulness, or vocalization can also be observed in other contexts outside conspecific death (e.g., distress from noises or nociceptive pain). This highlights the importance of cautious interpretation of animal responses. However, these behavioral changes indicate that a permanent loss of a social bond can trigger an emotional reaction that elicits them. That.

#### 3.1.1 Thanatological behaviors

In elephants, thanatological behaviors include mobbing, transport, removal, burial, ceremonial gatherings, cacophonous aggregations, alarm calls, and other vocalizations (106). They also consider dead infant carrying, aggression, vigils/guarding, cautious inspection, curiosity/approach, and visitations. In species such as free-ranging Asian elephants (*Elephas maximus*), post-mortem interactions have been reported by Sharma et al. (116), who observed approach, sniffing, and inspection responses, as well as epimeletic behaviors or physical support of dying calves in pre- and perimortem phases. In addition, high-frequency vocalizations (trumpets) were performed by an adult female in the presence of a dying calf. Although elephant trumpet blows have been classified as vocalizations related to social interaction with humans and conspecifics (117), Pokharel et al. (118) noted that calls directed toward the dying member or to human presence are frequently observed in Asian elephants in the presence of a dead calf.

In chimpanzees, Harrod et al. (119), through a comprehensive review of primatology reports, discussed the presence of thanatological behaviors such as silence (in groups or individuals), high-ranking individuals guarding corpses, solemn visits with sniffing, inspection, gazing upon the corpse, hair bristling and charging displays, distress calls, and departure behaviors. This reveals complex patterns in response to death. In this sense, multiple populations of chimpanzees, both wild and captive, have exhibited some of these behaviors, including those reported at the Arnhem Zoo by Whaal et al. (120), in female chimpanzees. Chimpanzees wail, whimper, and burst out screaming after the death of an infant. Moreover, after the sudden death of another female in the colony, her companion emitted non-threatening screams as a form of communication, which were answered by another female in a different part of the enclosure, triggering silence in the area. Similarly, in another case of a male dying in a fight, it was observed that when the body was left in the cage, the colony remained in absolute silence throughout its presence, even the following morning during feeding time. Vocal activity resumed when the body was removed.

Bekoff et al. (121) reported that after the death of a male chimpanzee in the New York University laboratory, other chimpanzees from the colony were allowed in to monitor their behavior. The animals exhibited behaviors such as tugging their arms, trying to open their eyes, grooming, and rubbing their bellies, while others wandered off hooting. This latter sound turned into screams and pounding on the walls of the enclosure. In the case of the natural death of elderly chimpanzees (40), observed on a UK safari, behaviors included pre-death care, such as remaining still, preparing the sleeping site, bringing food, touching, stroking, and grooming. Other peri-death behaviors included trying to find signs of life on the body, silence, viewing, sniffing, tugging arms, and mouth opening. Additionally, before and after death, there were displays of male aggression (swaggering, hair bristling, and running past the body), including charging displays where the surviving male struck the torso of the corpse with his fists. Other behaviors include returning to the body after death, silent watching, touching, reassurance grooming, and cleaning away straw. Alarm calls were also present, followed by post-death group lethargy.

Cronin et al. (122) also monitored the behaviors of a chimpanzee after the loss of her infant in a sanctuary located in Zambia, where reports mention that she inspected the face (16.5 s), touched the neck (5.4 s), face (5.1 s), body (2.5 s), gaze (6.7 s), peer (7.7 s), hand above infant’s face (2.4 s), fly swat (<1 s), and pick up infant (4 s). These behaviors, expressed by the mother upon the separation from her infant due to death, provide valuable insight into the chimpanzee response to the premature severing of the mother-infant bond, the maternal contribution to this bond, and how these animals gather information. These behaviors have been interpreted as thanatological responses, although their emotional meaning remains uncertain.

Despite the richness and diversity of thanatological behaviors described across species—from epimeletic care and corpse carrying to silence, vocalizations, and aggression— the role of neural circuits (including the periaqueductal gray, amygdala, and opioidergic systems) mediating separation distress and psychological pain related to the loss of a conspecific is still under research in non-human mammals.

### 3.2 Separation anxiety

Separation anxiety has been described as a set of behavioral and physiological responses associated with close attachment figures and is similar to separation anxiety disorder (29). Neurobiologically, animal models of separation stress have shown increased activity in prefrontal-amygdala circuits, suggesting a neural basis for attachment-related behavior (123). In this regard, Demarchi et al. (124) evaluated the neuronal activity from suckling Sprague–Dawley rats after the loss of their pup following birth. Obtained blood, brain, and tissue samples. The results indicated the impact of litter loss on neuronal activity from day one, showing an increase in the percentage of c-Fos/NeuN immunoreactivity in PL (layers II/III) compared to females housed with their litter (4.903% vs. 0.828%; *p* = 0.0003). Similarly, neuronal activity in the amygdala increased in the same groups (0.587% vs. 0.120%). These results indicate a positive linear relationship between PL and BLA neuronal activities in mothers when separated from their pups.

### 3.3 Broken heart syndrome (Takotsubo)

In humans, broken heart syndrome, takotsubo, ampullary cardiomyopathy (125), and apical ballooning syndrome (126) refer to a stress-induced cardiomyopathy described by the American Heart Association (127). The etiology suggests that intense emotional stress has been associated with autonomic dysregulation and stress-induced cardiomyopathy (24, 128). The incidence of anxiety and depression in this syndrome is between 21% (129) and 40% (130). The pathogenetic mechanisms of this syndrome directly involve catecholamine toxicity, microvascular spasm, and sudden myocardial stunning (131).

An animal model of takotsubo has been developed in cynomolgus monkeys through the application of repeated intravenous cardiac infusion of epinephrine overdose, which induced myopathy, with progressive left ventricular systolic dysfunction and depression of systolic function with severe hypokinesis in apical regions and hyperkinesis in the basal region (132). The study demonstrated that metoprolol reduced epinephrine-induced cardiomyocytolysis, thereby supporting the effectiveness of beta-blockers in the pathogenesis of the syndrome.

Ueyama et al. (23) used a murine model to reproduce the electrocardiographic and ventriculographic changes observed in takotsubo cardiomyopathy in postmenopausal women by employing a stress-immobilization model, which that activates the hypothalamic–pituitary-adrenocortical (HPA) axis and the sympathoadrenal system, simulating an emotional stress model. The study results reported that estrogen supplementation attenuated stress-induced sympathoadrenal outflow from the brain to the heart through direct action on the nervous system, thereby increasing the regulation of cardioprotective substances such as atrial natriuretic peptide and heat shock protein 70 (HSP70), which act directly on the heart. This suggests the potential benefit of estrogen in reducing the incidence of this cardiomyopathy. This syndrome exemplifies the interaction between emotional stress and cardiac dysfunction. Findings from animal models are currently helping elucidate the disease etiology and improve clinical management. Furthermore, they clarify that emotional stress not only has behavioral and neuroemotional consequences but also direct somatic effects.

## 4 Ethological manifestations and diagnostic indicators

**Figure 5:**
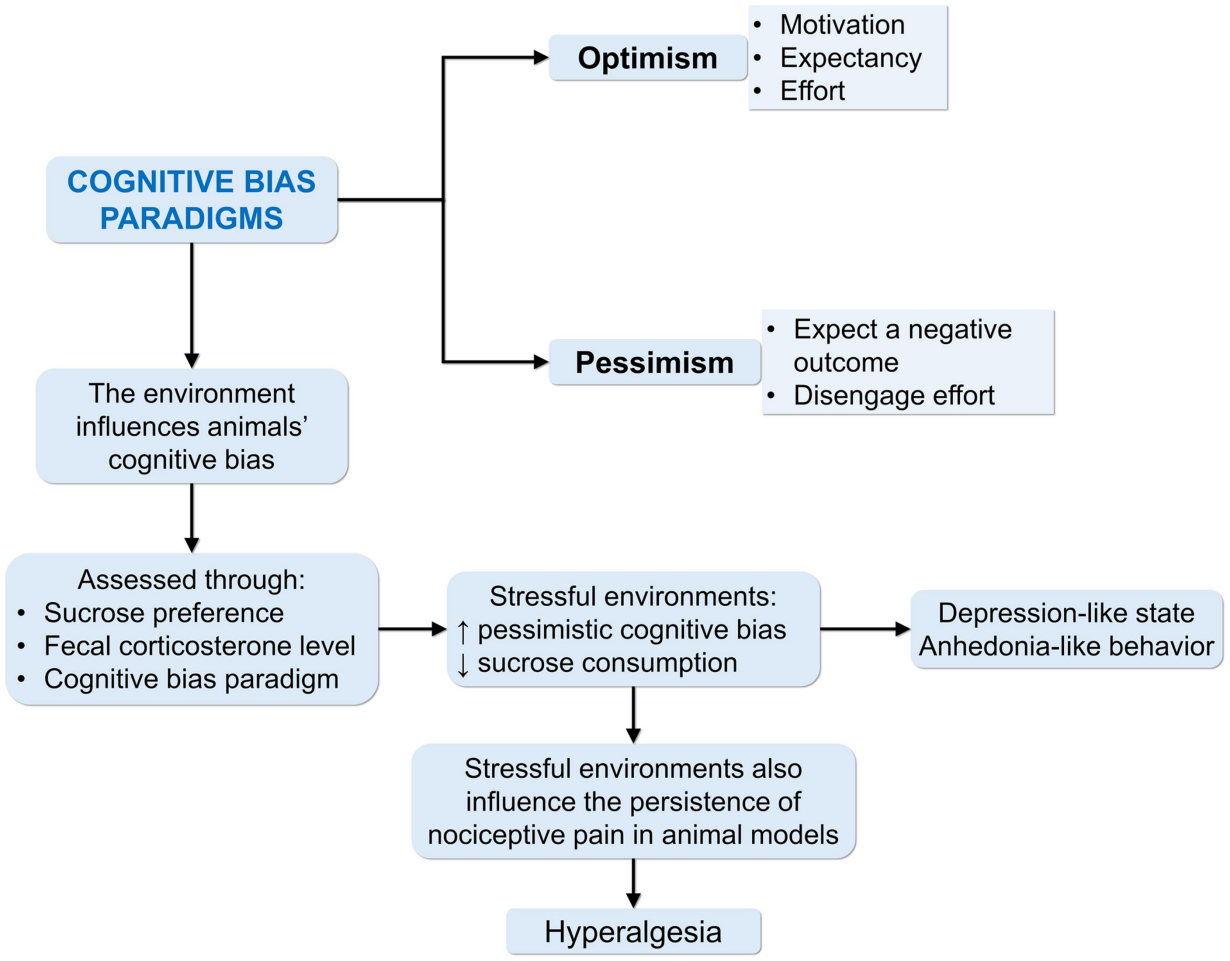
Indirect indicators of affective state in non-human animals. Cognitive bias paradigms as a method to assess the affective state of animals.

Measuring the affective state of non-human animals is challenging, as they cannot verbally convey it. To date, accurate methods to diagnosing or identifying emotional pain in non-human animals have not been established. However, researchers have adopted the so-called judgment bias paradigm from human medicine to understand how animals perceive their environment (133, 134). Cognitive bias paradigms are used as indirect indicators of affective state, based on how animals interpret ambiguous stimuli as positive or negative (133, 135). Optimism is related to motivation, expectancy, effort, and more optimistic judgments (134, 136). In contrast, pessimistic individuals expect a negative outcome and tend to disengage effort, reflecting their negative affective state. Figure 5 illustrates the cognitive bias paradigm as an indirect indicator of affective states in non-human mammals (134, 136, 137).

To determine whether an animal is optimistic or pessimistic, they are trained to respond to positive/negative conditions that involve a food reward, thereby assessing their emotional state (134). For instance, Rygula et al. (136) evaluated optimistic and pessimistic traits in male Sprague Dawley rats (175–200 g). The authors used an ambiguous-cue interpretation (ACI) paradigm to examine the correlation between these traits and motivation. Rats learned to associate a positive tone (2000 Hz at 75 dB) with a reward (5% sucrose solution), and a negative tone (9,000 Hz at 75 dB) with a forthcoming punishment (0.5 mA, 10 s). When assessing the average cognitive bias index, optimistic rats recorded indices ranging from 0.01 to 0.38, whereas pessimistic rats recorded indices ranging from −0.01 to −0.67. These indices reflect the affective state of animals, as individuals in negative affective states judge certain stimuli more negatively (138), which suggests that rats with the optimistic trait are more motivated to obtain the sucrose reward (136). This was also reported in another study by the same authors, where the sensitivity of optimistic/pessimistic male Sprague Dawley rats to positive/negative feedback (175–200 g) was assessed using the same methods (139). After classifying animals, it was found that optimistic rats recorded an index between 0.05 and 0.89. Conversely, pessimistic rats had a cognitive bias ranging from −0.01 to −0.65, and these animals responded significantly more often to the negative condition (a proportion of 0.6 *vs*. 0.3). These results are interpreted as reflecting differences in affective state or motivational processing.

In veterinary medicine, studies that correlate cognitive bias with emotional pain have not yet been published. However, in human medicine, it has been reported that pessimism significantly covaries with nociceptive pain intensity in patients with chronic low back pain (*F* = 14.5, *p* < 0.001) (140). Likewise, an optimistic trait has also been associated with a positive effect on pain perception (141). In human patients with acute and chronic nociceptive pain related to musculoskeletal disorders, arthritis, post-surgical, or cancer, optimism is associated with less pain intensity (142). In humans, optimism and pessimism have been associated with differences in pain perception, which suggests that affective state may influence nociceptive processing. Whether similar mechanisms are involved in animals remains unclear.

Studies have also shown the influence of the environment on animals’ cognitive biases, which is relevant for e individuals reared in negative environments, in whom distress might have implications for their affective state. Animals exposed to stressful environments showed a more pessimistic cognitive bias, which has been interpreted as an indicator of a negative affective state. In this sense, Barker et al. (135) compared caging conditions and the optimistic/pessimistic response of 3-week-old male and female Sprague–Dawley rats. The conditions were either standard cages or housing in metabolic cages (considered a stressful condition). By assessing sucrose preference, fecal corticosterone levels, and cognitive bias, the authors determined the effect that the environment has on rats’ optimistic or pessimistic decisions. It was found that animals housed in the metabolic cage exhibited significantly lower sucrose consumption (70 and 80%) than in standard cages (90%). Although no differences were recorded in fecal corticosterone, rats housed in metabolic cages exhibited a significantly greater pessimistic bias, and females had a significantly reduced number of days with an optimistic decision (control rats: up to 5 days vs. metabolic: less than 1 day). Reduction in sucrose consumption has been reported in rats subjected to chronic unpredictable mild stress paradigms (143). Similarly, in horses displaying withdrawn states and low responsiveness to their environment, low sucrose and hay consumption were reported (144), which are associated with distress.

These results suggest that animals can display a depression-like state (or pessimistic shift in cognitive bias), which can be influenced by the environment (135). For instance, Drozd et al. (137) determined, in male Sprague Dawley rats (175–200 g), the relation between cognitive judgment bias and stress-induced cognitive inflexibility. After two weeks of handling stress (isolated and transferred to individual metabolic cages), it was found that pessimistic rats had a significantly longer latency to approach the reward than optimistic conspecifics (up to eight seconds).

Studies focusing on optimistic and pessimistic animals have also highlighted the role of anhedonia-like behavior in animals’ biases. Anhedonia is a condition characterized by “the decreased ability to experience pleasure from positive stimuli or a degradation in the recollection of pleasure previously experienced” (145). In humans and non-human animals, anhedonia is markedly observed by a reduced interest or pleasure in previously enjoyable activities (146). This condition could be related to emotional pain, as studies have shown that long-term exposure to aversive conditions triggers affective disorders (146, 147).

The sucrose preference test has been established as a widely used and reliable experimental model of anhedonia in nonhuman animals (148). Reduced sucrose preference is widely used as a behavioral marker of anhedonia in animal models (147). For example, Rygula et al. (149) determined that “pessimist” animals are susceptible to stress-induced anhedonia. In the study, male Sprague–Dawley rats (175 and 200 g) were subjected to chronic restraint to evaluate the influence of optimistic/pessimistic traits on sucrose preference. The cognitive bias index for pessimistic rats was −0.6, while stressed optimistic rats had indexes up to approximately 0.4, suggesting that chronic stress makes rats more pessimistic. For both traits, chronic stress induced anhedonia-like responses. However, the effects were significantly longer in pessimistic rats, who significantly reduced their sucrose consumption. This suggests that prolonged periods of stress can induce affective deficits in animals. This was also reported in naïve Wistar male rats (180–200 g) subjected to chronic social stress (147). Chronically stressed rats displayed behavioral alterations related to depressive-like responses. These alterations included a significant decrease in locomotor activity (to 4,000 counts/10 min) and a significant increase in immobility in the forced swim test (to above 200 s during the five-minute test). Additionally, sucrose consumption decreased in stressed rats (55%) when compared to non-stressed animals (75%).

The association between an animal’s affective state and emotional pain remains unclear. However, studies have shown that anhedonia-like states and negative emotional states are related to the persistence of nociceptive pain. In this context, a rat model of endometriosis found that water-avoidance stress induced hyperalgesia (150). Similarly, Cao et al. (151) reported that chronic stress increased pain sensitivity in male and female Sprague–Dawley rats (200–300 g) after incisional surgery. In these animals, immobilization stress for 6 h over 3–5 days induced hypersensitivity, together with significant increases in serum corticosterone (up to 20 ng/mL), and decreases in sucrose consumption (by 50%). These findings suggest an interaction between stress, affective state, and nociceptive pain processing.

These responses are related to neural changes induced by nociceptive pain, as described by Thompson et al. (152) in male Sprague–Dawley rats (150–200 g) subjected to nerve injury. In these animals, chronic nociceptive pain significantly reduced sucrose preference, from 59.92 ± 3.04% to 58.88 ± 3.56%. Injured rats also had reduced opioid receptor availability in the insula, caudate–putamen, and motor cortex, reflecting alterations in the endogenous opioid system. Besides, it was reported that the expression of the *μ*-opioid receptor (MOR1) was significantly lower in injured animals in the caudate–putamen (receptor label intensity 8,437 ± 437 *vs*. 9,832 ± 358) and insula (8,862 ± 452 *vs*. 10,390 ± 398). Nociceptive pain also negatively affects goal-directed behaviors and motivation, as inflammatory pain has been reported to decrease dopaminergic neuronal activity in the ventral tegmental area, a critical region for motivational states (153). Chronic pain models have shown alterations in opioid and dopaminergic systems associated with reduced motivation and anhedonia-like behavior.

As assessing the emotional state of non-human animals is challenging, cognitive bias paradigms can indirectly provide an indicator of their affective state. Although there are no studies directly associating certain behavioral responses with emotional pain, identifying anhedonia-like and depression-like behaviors could suggest affective disorders.

## 5 Physiological and immunological consequences associated with emotional pain

**Figure 6:**
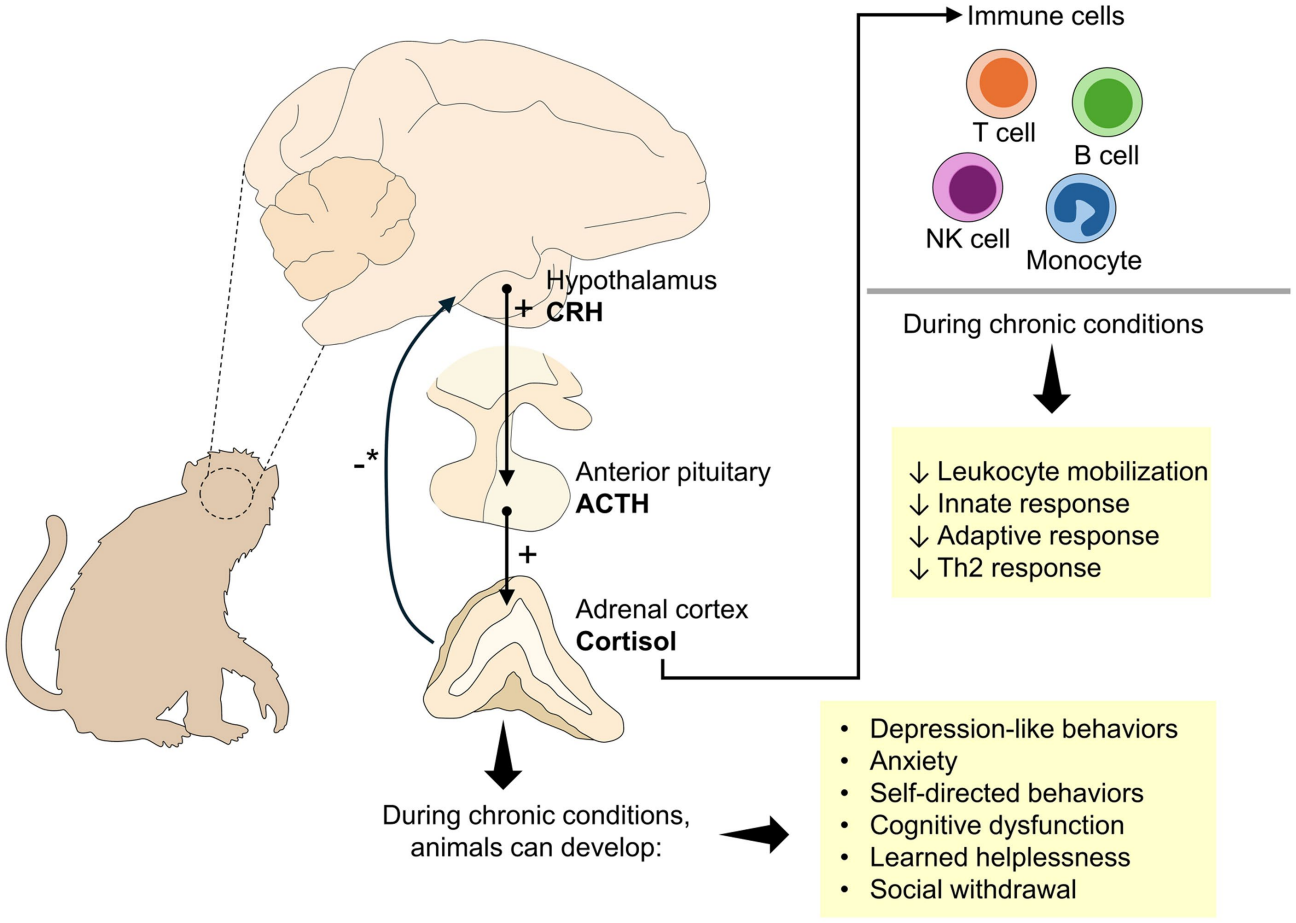
Neuroendocrine and immunological consequences associated with persistent emotional pain. Persistent emotional pain engages neural circuits that integrate the affective–motivational dimension of pain, including the amygdala–parabrachial pathway, which modulates both pain perception and its emotional valence. Dysfunction of this pathway during prolonged pain states promotes neuronal hyper-excitability and amplifies aversive experience. Concomitantly, sustained activation of the hypothalamic–pituitary–adrenal (HPA) axis leads to the release of glucocorticoids, which regulate bidirectional communication between the central nervous system and the immune system. At the immunological level, emotional pain is associated with alterations in the adaptive immune response, reflected by changes in the proliferation, differentiation, and function of T and B lymphocytes, as well as the activation of specific subpopulations such as T helper 17 (Th17) cells, which have been implicated in the pathophysiology of depressive disorders. These neuro-immune–endocrine interactions promote the persistence of negative affective states, accompanied by immune dysregulation and depression-like behaviors (154, 158, 159).

Maladaptive neuroplasticity and altered excitability in the neurocircuitry involved in the emotional-affective component of pain are linked to an imbalance in the adaptive immune cell population in models of persistent pain (154). Alterations in the relative abundance of lymphocyte have been observed in patients with chronic pain, consistent with an abnormal response to endogenous glucocorticoids (154–156). Specifically, depression disorders, chronic stress, and neuropathic pain may contribute to eliciting alterations in the expression of corticotropin-releasing factor (CRF) receptors. For example, Kiritoshi et al. (155) showed that the spino-trigemino-parabrachio (PB)-amygdaloid pathway increases the hyperexcitability of CRF projection neurons in mice during neuropahtic pain induced by spinal nerve ligation surgery, compared with

control mice. Thus, the spino-trigemino-PB-amygdaloid pathway appears to play a crucial role in the somatosensory and emotional-affective aspects of pain (157), in which elevated CRF expression may reflect a state similar to chronic stress potentially leading to immune dysregulation as a result of persistent pain. Thus, during acute pain, the glucocorticoid system acts as a negative feedback mechanism on the HPA axis and promotes the proliferation, differentiation, and recruitment of immune cells including leukocytes, T and B cells, neutrophils, among others. However, under conditions of chronic stress (induced by the high-dose exogenous glucocorticoids or prolonged exposure to moderate corticosterone) (154, 158), reduced immune response is observed. It is associated with depressive-like and anxiety-like behaviors, as well as reduced leukocyte trafficking, neutrophil phagocytosis, and a reduction in peripheral lymphocytes (Figure 6) (154, 159).

T and B cells, primary components of the adaptive immune response during infections, may attenuate their immune activity at high corticosteroid concentrations re (160). In male C57BL/6J mice subjected to chronic stress via social defeat stress, it was observed that, on the tenth day of consecutive social defeats, the concentration of T cells was significantly lower (13%) than in mice under non-stressful conditions (25%) (161). This may be associated with glucocorticoid effects on receptors expressed in T cells. Elevated concentrations of glucocorticoids are consistent with suppression of the activation of cell lineages responsible for the adaptive immune response and with apoptosis in peripheral T cells (154). Therefore, abnormal CRF expression due to persistent pain states leads to elevated cortisol concentrations, which, in turn, promote chronic stress and may lead to dysregulated adaptive immune response. It is worth noting that there is reciprocal feedback between the glucocorticoid system and the HPA axis; thus, dysfunction in the HPA axis during poorly tolerated or excessively stressful events may represent a disproportionate release of stress hormones, resulting in persistent aversive affective states (e.g., chronic pain, depression-like disorders) at the brain level, and suppress immunity (e.g., inhibited release of T and B cells) (155).

This condition, similar to chronic stress, depression disorders, and persistent pain, has also been observed in other centers involved in emotional mechanisms, such as the hippocampus. In this case, the hippocampus has a neuronal population that expresses the highest concentrations of CRF receptors in mammals, and maintain extensive connections with the ACC and BLA. Recent studies have shown that under pathophysiological conditions, such as chronic pain, a state similar to chronic stress is induced due to elevated expression of cortisol-sensitive genetic markers (162). Another possible explanation involves glucocorticoid-suppressed T cell lineages and the favoring of a particular lineage, Th17 cells. T helper (Th17) cells are closely related to the manifestation of depressive behaviors. In general, chronic stress resulting from elevated CRF expression in the brain may lead to the pathogenic activation of T helper (Th) cells. This cell lineage reactivate metabolic pathways that deplete tryptophan availability, a precursor molecule for serotonin synthesis (154).

In this regard, it is also suggested that a shortage of neurotransmitters such as serotonin could mediate the manifestation of depressive-like behaviors. Therefore, these adverse effects underscore the severity of depressive disorders and their undeniable impact on the affective and immunological state of patients. Although the association between emotional pain and immune system responses remains unclear, the mechanisms observed in chronic stress models, persistent pain, and depression-like states appear to be a key component involved in dysregulation of the adaptive immune response in prolonged affective negative states. From this broader perspective, neuroendocrine and immune responses associated with negative affective states should not be regarded as processes isolated from the behavioral and expressive changes accompanying emotional experience. In this context, changes in facial expression may reflect the integration of affective and motor circuits—including the amygdala, cortical regions, motor pathways, and the facial nerve—and could provide complementary, non-invasive indicators of pain-related responses and other negative emotional states, while requiring careful consideration of species-specific differences (163, 164).

## 6 Conclusion

Psychological or emotional pain has been mostly studied in humans. Conversely, in non-human animals, research is still ongoing, as understanding animal emotions is challenging. However, in humans, it has been shown that chronic emotional pain could be involved in emotional disorders such as dysthymia, grief, and Takotsubo cardiomyopathy, among others. These disorders result from the convergence of distributed corticolimbic circuits that play a key role in the management of physical and psychological pain. Affective dysregulation depends not only on the activity of fear centers but also on the collapse of the cortical inhibitory system, whose imbalance between excitation and inhibition prevents the extinction of emotional pain. The loss of social bonds in non-human animals activates complex emotional, physiological, and behavioral changes such as grief-like responses and separation-related distress that can lead to prolonged affective stress. Despite growing evidence, the main limitation of the study of emotional pain in non-human animals is that many neurobiological mechanisms have been inferred from chronic stress or nociceptive pain models. This highlights the relevance of these findings for future comparative research aimed at expanding our understanding of emotional pain in non-human animals.
